# fMRI-Indexed neural temporal tuning reveals the hierarchical organsiation of the face and person selective network

**DOI:** 10.1016/j.neuroimage.2020.117690

**Published:** 2020-12-29

**Authors:** Silvia Ubaldi, Scott L. Fairhall

**Affiliations:** Center for Mind/Brain Sciences (CIMeC), University of Trento, Corso Bettini 31, Rovereto, TN 38068, Italy

**Keywords:** Network-dynamics, Face perception, Place perception

## Abstract

Recognising and knowing about conspecifics is vital to human interaction and is served in the brain by a well-characterised cortical network. Understanding the temporal dynamics of this network is critical to gaining insight into both hierarchical organisation and regional coordination. Here, we combine the high spatial resolution of fMRI with a paradigm that permits investigation of differential temporal tuning across cortical regions. We cognitively under- and overload the system using the rapid presentation (100-1200msec) of famous faces and buildings. We observed an increase in activity as presentation rates slowed and a negative deflection when inter-stimulus intervals (ISIs) were extended to longer periods. The primary distinction in tuning patterns was between core (perceptual) and extended (non-perceptual) systems but there was also evidence for nested hierarchies within systems, as well as indications of widespread parallel processing. Extended regions demonstrated common temporal tuning across regions which may indicate coordinated activity as they cooperate to manifest the diverse cognitive representation accomplished by this network. With the support of an additional psychophysical study, we demonstrated that ISIs necessary for different levels of semantic access are consistent with temporal tuning patterns. Collectively, these results show that regions of the person-knowledge network operate over different temporal timescales consistent with hierarchical organisation.

## 1 Introduction

Effective interpersonal interaction is fundamental to daily life. To accomplish this, we need not only to perceive and recognise conspecifics but also to know things about them. Such ‘person-knowledge’ incorporates personal traits, intentions, attitudes, biographical information and episodic memories related to specific individuals. This knowledge is served by a well-characterised neural network classically divided into core and extended systems (Fairhall and Ishai, 2007; Gobbini and Haxby, 2007; Haxby et al., 2000).

The core system is closely related to the perception of faces and includes occipital/fusiform face areas (O/FFA) and posterior superior temporal sulcus (pSTS; Haxby et al., 2000). The extended system, more active when viewing familiar people, is thought to be involved in spontaneous access to person-knowledge (Gobbini et al., 2007; Haxby and Gobbini, 2011) and is recruited by both names and faces (Fairhall et al., 2014; Fairhall and Caramazza, 2013; Tempini et al., 1998; Wang et al., 2016). The extended system includes the amygdala, inferior frontal gyrus (IFG), medial prefrontal cortex (mPFC), precuneus, and the anterior temporal lobes (ATL; Gobbini et al., 2007).

It remains unclear how person-knowledge is organised within this system. The seminal model of Bruce and Young (1986) posited serial access to successive stages of person-knowledge while allowing parallel access to different kinds of person-knowledge (Burton and Bruce, 1992). This model is generally supported by behavioural evidence. Serially, basic category distinctions, such as faces vs. vehicles, are made as fast as 150 msec with saccades (Crouzet and Thorpe, 2011) or 260 msec with manual responses (Barragan-Jason et al., 2012; Rousselet et al., 2003), while familiarity judgments are relatively slower at 500-600 msec (Barragan-Jason et al., 2012; but see Visconti di Oleggio Castello and Gobbini, 2015) and are followed by broad semantic categorisation at 600 msec (e.g. occupation; Schweinberger et al., 2001). Subsequent access to more complex knowledge, such as a politician’s political party (667 msec), has a time course parallel with nominal information (first letter of name, 671 msec; Abdel Rahman et al., 2002). Electrophysiological evidence also points to varying timescales for access to different forms of information. Reliable correlates of face familiarity emerge with the temporal-occipital N250 component (Schweinberger et al., 2002; Tanaka et al., 2006). Multivariate analyses of MEG data indicate that, while representations of face familiarity emerge with a latency of 400 msec, differential representations of face identity are potentially more strongly pronounced for familiar than unfamiliar faces as early as 100 msec after stimulus onset (Dobs et al., 2019). These early responses may however reflect image-based properties, with identity representations that are tolerant to variations in image properties and insensitive to face-gender emerging at 400 msec (Ambrus et al., 2019). Later N400 and P600 components have been associated with semantic access (Bentin and Deouell, 2000; Eimer, 2000a, Eimer, 2000b; Gosling and Eimer, 2011; Henson et al., 2003). These behavioural and electrophysiological techniques provide insight into the hierarchical organisation in face processing but lack the spatial acuity to reconcile findings with the fMRI-delineated person-knowledge network.

**Fig. 1:**
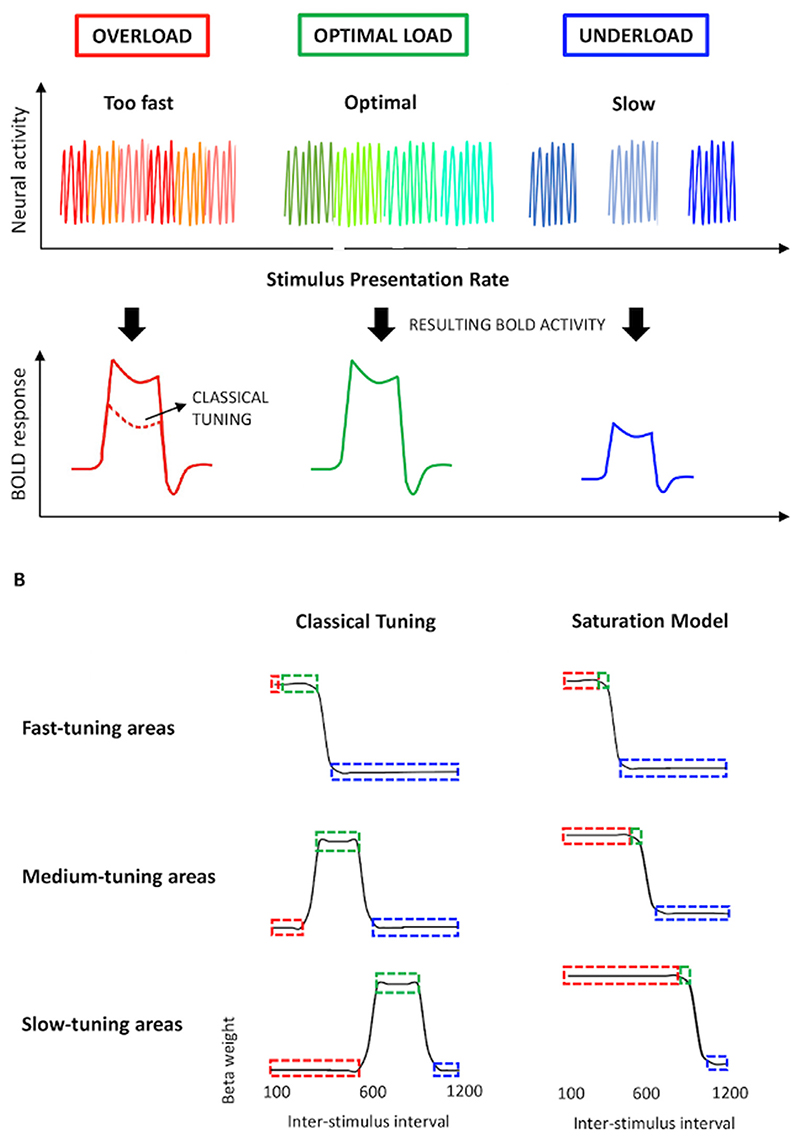
Schematic illustration of predicted model of cortical temporal tuning. The upper part of panel A represents hypothetical neural activity produced by stimuli presentation at fast, medium and slow inter-stimulus intervals. Neural activity is modelled as the response to the stimulus until interrupted by neural activity arising from the subsequent stimulus. The bottom part of panel A depicts the resulting BOLD activity for each presentation rate across an fMRI block. Panel B represents the translation of the block fMRI response into a tuning function. This is accomplished by plotting the average block fMRI response as a function of ISI. This approach will produce different tuning curves in brain regions operating across different timescales. Shown are hypothetical tuning curves for fast-, medium- and slow-tuned cortical areas, based on either the saturation model (left) or classical tuning model (right). Here, ‘classical tuning’ is used to refer to a model predicting reduced fMRI response when the region is overloaded due to temporal processing capacity limits, in addition to the signal reduction during underloaded conditions that is predicted by both models.

Our goal was to combine the high spatial resolution of fMRI with a paradigm that permits investigation of the temporal-tuning properties of brain areas. We manipulate rates of famous face presentation over twelve inter-stimulus intervals (ISIs) ranging from 100 to 1200 msec. We rely on the properties of neural-haemodynamic coupling to make inferences about the temporal tuning of different brain regions by averaging the signal over the block (and translating duration into amplitude; Fig. 1). Specifically, at presentation rates slower than the region’s time-constant, it can be thought of as being under-loaded –the execution and completion of its operation will be followed by a period of neural idleness. Over an fMRI block, alternating active and idle periods result in a relative amplitude reduction in the fMRI response. Increasing presentation rate will increase a brain region’s ‘time-on-task,’ and thus the amplitude of the block fMRI response, until it matches the region’s time-constant. A region’s time-constant can be thought of as the time taken for its operation/computation to complete. At this optimal load (Fig. 1, green), neural activity is constant.

At presentation rates faster than a region’s time-constant, we could expect a diminished response consistent with a ‘classical’ tuning function, similar to that of the single unit (Fox and Raichle, 1985; Singh et al., 2000). In this case, a region will also respond less at rates faster than its optimal presentation rate (Fig. 1, classical tuning in red). Such reduced neural activity is consistent with investigations of temporal tuning in the visual system (Gauthier et al., 2012; McKeeff et al., 2007; Stigliani et al., 2015), and may reflect endogenous limits in a region’s temporal processing capacity or that hierarchically upstream neural processes have not had sufficient time to process the stimuli. Alternatively, at presentation rates faster than the specific region’s time-constant, the neural response may ‘saturate’ as the incomplete but continuous processing of incoming stimuli translates to a saturated haemodynamic response (Fig. 1, saturation model in red).

We predict that different regions will have different tuning, with faster time-constants associated with simpler cognitive operations and longer time-constants in regions underlying higher-level functions, and hypothesise that regions with similar functional roles will demonstrate similar temporal-tuning profiles.

## 2 Materials and methods

### 2.1 Participants

Forty-six healthy volunteers (mean age 22.6, 15 males) participated in the study. All participants were right-handed native Italian speakers. Participants had no history of neurological disorders and had normal or corrected-to-normal vision. All participants gave informed consent to take part in the study and were reimbursed for their time. Procedures were approved by the Ethical Committee of the University of Trento.

### 2.2 Stimuli

**Fig. 2:**
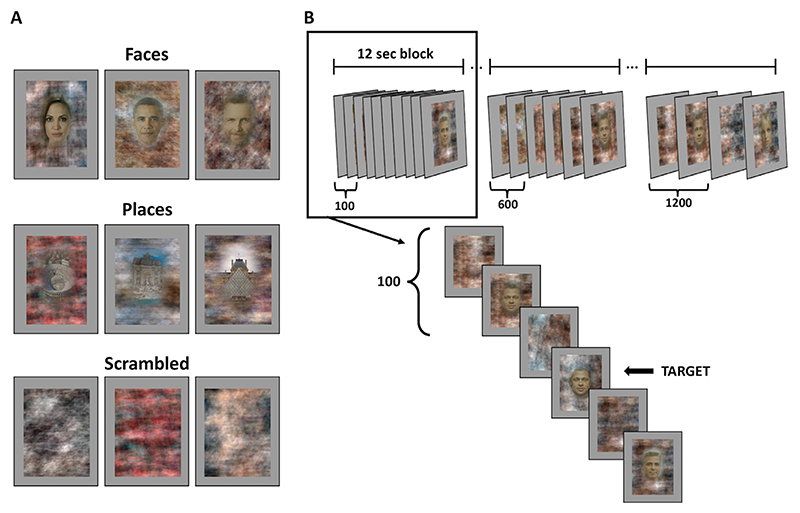
Experimental design. Panel A. Examples of experimental stimuli for the three categories: faces, places and scrambled. Panel B. Each experimental run was divided into 28 blocks: 12 faces, 12 places and 4 scrambled. Each block lasted 12 secs and had a specific ISI, from 100 to 1200 msec (in steps of 100 msec).

Stimuli were 54 Italian and foreign celebrities, amongst which were actors, singers, entertainers, politicians and sportspeople, and 52 famous monuments from around the world. We selected stimuli from three different pictures of each face identity. Stimuli were presented inside an oval shape superimposed on a phase-scrambled background (Fig. 2). The faces were all in frontal view with a neutral expression, and all the stimuli were matched for luminance and dimension on the screen. For the baseline condition, we used 24 phase-scrambled images. Stimuli (600 ×800 pixels) were presented with Psychtoolbox (Brainard, 1997; Pelli, 1997) running on MATLAB (Mathworks).

### 2.3 Experimental paradigm

The experiment was split into 4 fMRI runs. Each run was composed of 12 randomised blocks of famous face stimuli, 12 blocks of famous place stimuli and 4 blocks of scrambled images (Fig. 2). Stimuli were presented at 12 different ISIs (one ISI per block), ranging from 100 to 1200 msec in 100 msec steps (see Fig. 2). Face and place stimuli were presented for half the ISI followed by the phase scrambled background alone for the other half. In this way, the total time an object (face or place) was onscreen was balanced across the different ISIs. Scrambled images appeared every seventh randomized block and had a fixed ISI of 1 second. Each block lasted 12 s and blocks were separated by a 2-second fixation cross.

Participants performed a one-back matching task for each condition: they responded via button box with their right index finger when they saw the mirrored repetition of the same image (average one per block, ± 1). In face and place conditions, the target image had a different phase-scrambled background to avoid visual facilitation. After the scanning session, participants were asked which famous people they recognized, and which they had seen for the first time. All the participants considered in the analysis had a recognition accuracy above 90%.

### 2.4 fMRI data acquisition

Functional and structural data were collected with a Bruker BioSpin MedSpec 4-T scanner (Bruker BioSpin GmbH, Rheinstetten, Germany) while participants lay in the scanner and viewed the visual stimuli through a mirror system. Data collection was conducted at the center for Mind/Brain Sciences (CIMeC), University of Trento, using a USA Instruments 8-channel phased-array head coil. Functional images were acquired using echo planar (EPI) T2 * -weighted scans. Acquisition parameters were: repetition time (TR) of 2 s, an echo time (TE) of 33 ms, a flip angle (FA) of 73°, a field of view (FoV) of 192 mm, and a matrix size of 64 ×64. Total functional acquisition consisted of 796 vol over 4 runs, each of 34 axial slices (which covered the whole brain) with a thickness of 3 mm and gap of 33% (1 mm). High-resolution (1 ×1 ×1 mm) T1-weighted MPRAGE sequences were also collected (sagittal slice orientation, centric phase encoding, image matrix = 256 × 224, field of view = 256 × 224 mm, 176 slices with 1-mm thickness, GRAPPA acquisition with acceleration factor = 2, duration = 5.36 min, repetition time = 2700, echo time = 4.18, TI = 1020 ms, 7° flip angle).

#### 2.4.1 fMRI analysis

Data from thirty-five participants are included in this analysis. Data from eight subjects were rejected due to head motion (within-run movement greater than 2 mm). Two subjects were removed due to low target-detection scores (2.5 SD below the mean). One participant could not complete the test.

Data were analysed and pre-processed with SPM12 (http://www.fil.ion.ucl.ac.uk/spm/). The first 4 vol of each run were discarded. All images were corrected for head movement. Subject-specific parameter estimates (*β* weights) for each of the 24 conditions (faces and places for each ISI) were derived through a general linear model (GLM). Regressors were formed by convolving a box car function modelling each block with the standard SPM haemodynamic response function. A more lenient implicit masque for inclusion in the GLM (0.1 instead of the SPM default of 0.8) was coupled with an explicit grey matter masque to maximise sensitivity in susceptibility-sensitive regions on our 4T scanner. The control condition with scrambled images formed the implicit baseline. The 6 head-motion parameters were included as additional regressors of no interest.

#### 2.4.2 ROI selection

For consistency with previous research and generalisability with more standard presentation rates, initial coordinates for ROI selection were defined using group coordinates taken from an independent study (*N* = 44) in which subjects had to perform a one-back task on stimuli representing faces, objects and animals (faces > [*animals, objects*]). Specifically, for each subject, we identified the peak in the present experiment closest to those coordinates using the contrast faces > places at *p* < .005. For participants who did not have a peak within 10 mm, we reverted to the group coordinates at *p* < .005 (see Table 1 for coordinates). We then defined the conjunction of the subject-specific activation with a sphere of 6-mm radius around the subject’s peaks. The use of the orthogonal face > place contrast allows the identification of subject-specific face-selective voxels while protecting against erroneous differences between ISIs as a result of circularity (Friston et al., 2006). Beta values for each inter-stimulus interval were then extracted from these ROIs using MarsBar (Brett et al., 2002) and averaged across runs. In the complementary analysis for places, ROIs were identified with the contrast places > faces, as the coordinates for these regions were not available in the abovementioned dataset. Then we found subject-specific peaks of activation and extracted and averaged beta-values from voxels exceeding *p* < .005 within a 6-mm radius for each ROI and each ISI.

Proof-of-concept ROIs were created by marking the conjunction of the functional contrast: all images (faces and places) greater than baseline, and Brodmann regions 17, 18, 19 and 37 (as defined in the MRIcron template; https://people.cas.sc.edu/rorden/mricron/).

#### 2.4.3 Temporal-Tuning analysis

We used the same analysis for both face-selective and place-selective regions. After extracting the averaged beta value for each ROI and each ISI, to attenuate noise and attain a more reliable estimate of temporal tuning, we smoothed the data from single subjects across ISIs using a 3-point moving average, weighted with a kernel of [.25 5 0.25]. To assess tuning, we performed a one-way ANOVA, with a Greenhouse-Geisser correction (when applicable), to determine the simple effect of ISI, while an interaction between ISI and category was used to assess the specificity of the ISI effect to the category of interest. To assess differential tuning across regions for face stimuli, we ran a two-way ANOVA (ROI x ISI), to highlight the differences in the areas for the preferred category of stimuli. Then, in a pre-planned strategy to maximise power and sensitivity, we ran pairwise t-tests comparing ISIs 200 msec apart (e.g. ISIs 100 to 300, 200 to 400, 300-500 and so on).

To assess whether subdivisions of the network for perceiving and knowing about others cluster together in terms of their temporal-tuning profiles, hierarchical clustering analysis was performed across regions via Matlab. Dissimilarity in temporal tuning was quantified via correlation (1-r) for each subject. The results were then averaged across subjects and submitted to Ward clustering.

### 2.5 Psychophysical study

An independent sample of 27 subjects (7 male, mean age 24.5) performed 3 tasks in which they had to detect the consecutive presentation of two target stimuli, which were either males (gender), politicians (binary semantic category) or non-Italian actors (combinatorial category), amongst non-target distractors and target singletons. This strategy was chosen to more closely match our fMRI model and ensure full processing of the relevant dimension. The experiment was performed in 4 runs of 18 blocks collected over two separate days to avoid participant fatigue. Each block was composed of six trials (3-3.5 second) of a single combination of task and ISI. Six ISIs were selected along a logarithmic timescale (0.100, 0.167, 0.233, 0.367, 0.567 and 0.867) to maximise sensitivity across a range of ISIs while avoiding the need for an excessive number of trials. Participants indicated with a button press whether or not a target was present in the trial (50%) and received feedback on their performance.

To index ceiling performance for each task, a subset of 15 participants (5 male, mean age 25.0) performed an untimed version of the experiment (ISI≅inf). Subjects pressed a button for every image, indicating whether it was a target or non-target. Participants performed one run, divided into 12 blocks with 4 repetitions for each task. To ensure that sub-ceiling performance in this task was not due to participants being unfamiliar with famous identities, we collected recognition data at the end of the experiment.

## 3 Results

### 3.1 In-Scanner behavioural results

Repeated-measure ANOVA of accuracy, (for all reported ANOVAs, Greenhouse-Geisser corrections are implemented when Mauchly’s test indicated non-sphericity), showed an effect of Category (faces and places; F _(1,34)_ = 29.8, *p* < .0001), with face repetitions detected more accurately than places. A main effect of ISI (F _(7.5,254)_ = 69.2, *p* < .0001) motivated pairwise comparison that showed accuracy increased in a stepwise fashion from 100 msec, reaching an asymptote between 500 and 600 msec (~80% of accuracy). However, no significant interaction was observed between Category and ISIs (F _(6.8,233)_ = 2, *p* = .06), indicating that participants had a similar profile of performance across ISIs for both face task and place stimuli.

### 3.2 Proof of concept: temporal tuning in visual cortex

**Fig. 3:**
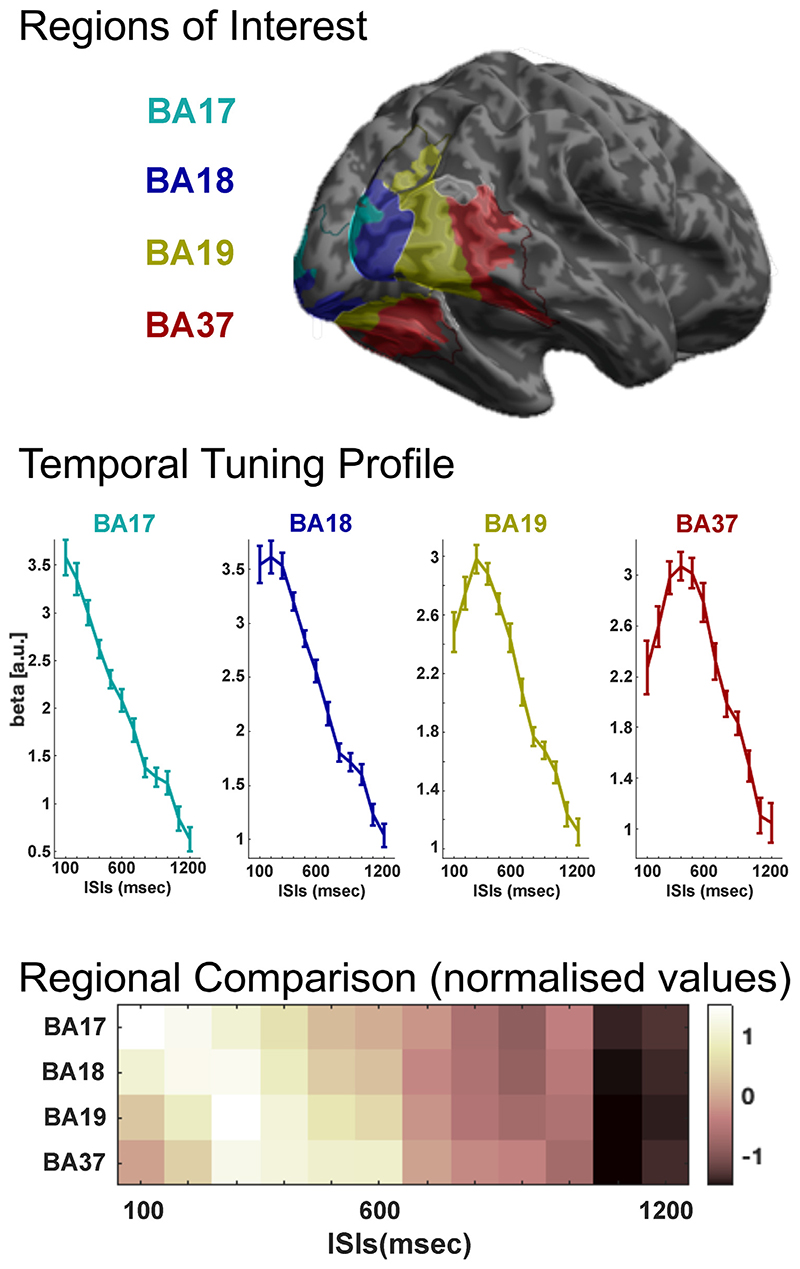
Temporal tuning in hierarchical visual cortex. Shown is the response to the presentation of images (both people and places) at different ISI (100-1200 msec), extending along the visual processing hierarchy. An increase in duration of temporal tuning can be seen across the visual processing hierarchy. The inflection points can be seen to occur at 100 msec in BA17, 300 msec BAs 18 & 19 and 400 msec in BA37, estimating that the underlying neural computations complete at this point. BA17 and 37 can be seen to show a reduced response at faster ISIs, consistent with a classical tuning model.

Firstly, to validate our overall approach and the viability of using differing ISIs to delineate temporal-tuning properties across cortical regions, we considered alterations in temporal tuning across the well-defined hierarchical layers of the visual cortex. To this end, beta values for the 12 different ISIs were extracted from stimulus-driven voxels (both faces and places, *P* < .001) in BAs 17, 18, 19 and 37 (see methods). Results are presented in Fig. 3.

We see clear evidence for tuning across the visual hierarchy. BA17, the cytoarchitectonic homologue of V1, showed rapid temporal tuning, responding maximally at the 100 msec presentation rate. BA18 showed tuning for longer presentation rates, with an inflection point at 300 msec, implying that at longer ISIs, the neural processing of the stimuli had sufficient time to initiate and complete and that progressively longer ISIs led to a relatively ‘idle’ period in the neuronal response between stimulus presentations. Temporal tuning was longer in BA19 and BA37, with an inflection point around 300 msec in BA19 and around 400 msec in BA37. Notably, at faster presentation rates, BAs 19 and 37 showed a relative reduction in response rate, consistent with the classic tuning model (c.f. Fig. 1).

The influence of ISI on response amplitude was formally assessed with a repeated measures ANOVA (main effect of ISI, all p-values < 0.0001). The difference in temporal tuning between regions was confirmed statistically via ANOVAs between each paired combination of regions (all p-values < 0.0001). ISI-by-Region interactions were performed using normalised (Z-transformed) data to ensure that positive effects were driven by differences in the true shape of the temporal tuning profile and not potentially because of apparent variations in shape arising from relative magnitude differences.

### 3.3 Category-Selectivity: whole-brain analysis

**Fig. 4:**
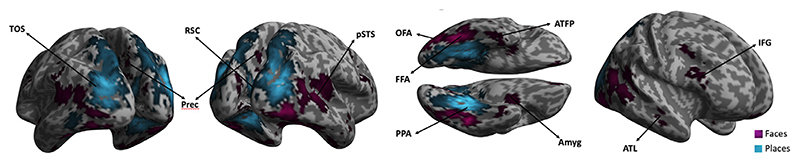
Category-selective response averaged across ISIs. In purple are face-selective areas: OFA, FFA, pSTS, IFG, Precuneus, ATL, ATFP, Amygdala. In blue are depicted place-selective regions: PPA, TOS and RSC (threshold: *p* < .001; extent: 30 voxels).

To be sure our rapid presentation paradigm produced activation broadly consistent with traditional, slower presentation rates, we also performed a standard whole-brain group analysis. The contrast faces-greater-than-places, collapsing across ISI, showed the expected activation of the core and extended systems (see Fig. 4, *p* < .001), although we did not find any active voxels in the angular gyrus at these relatively rapid presentation rates. The contrast places vs faces (*p* < .001), showed significant bilateral activation in place-selective regions (see Fig. 4): the para-hippocampal place area (PPA), the transverse occipital sulcus (TOS) and the retrosplenial complex (RSC; Epstein and Kanwisher 1998; Dilks et al., 2011).

### 3.4 Person-Selective regions

**Fig. 5:**
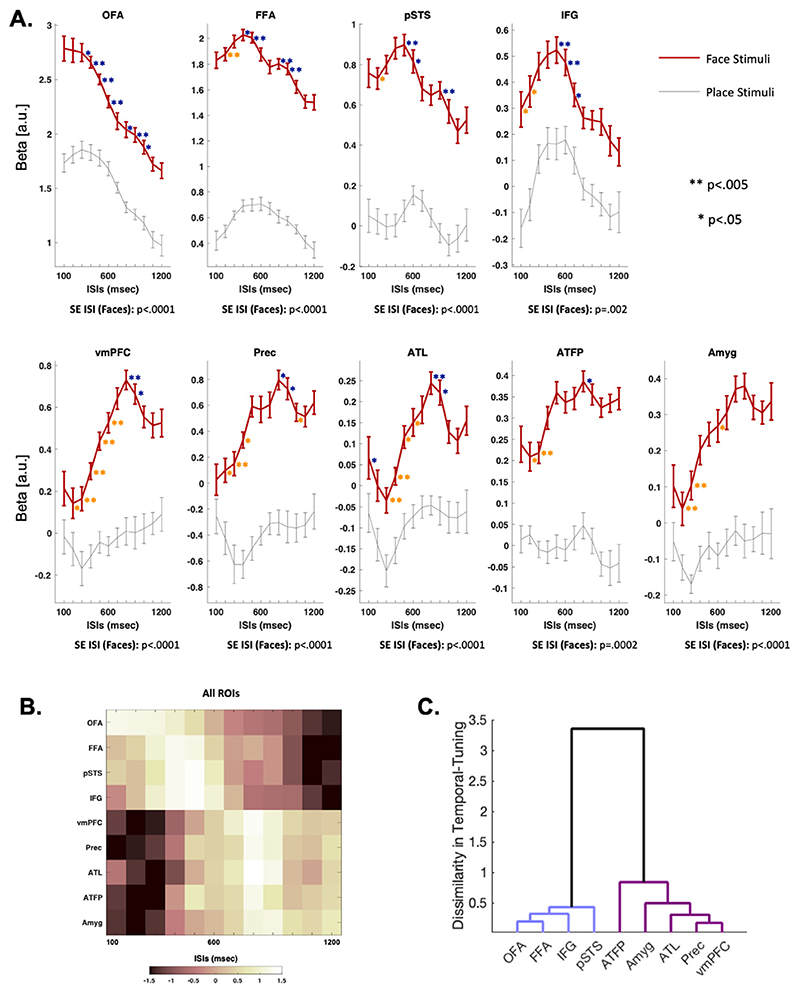
Temporal tuning in face-selective regions for face and place stimuli. A. Plots depict mean beta values (± 1 SEM within-subject; Cousineau, 2005) as a function of ISIs. *Upper:* temporal tuning of face stimuli in core system and IFG. OFA showed a temporal profile tuned to the fastest presentation rates, while other areas displayed slightly longer tuning, peaking between 500 and 600 msec. *Lower*: temporal tuning in extended system regions. These regions are strongly suppressed at faster presentation rates and show a maximal response at ISIs of 800 msec before displaying a reduction in responsivity as ISIs lengthen. Under each ROI are reported the Simple Effect (SE) of ISI. B. Comparison heatmap of temporal tuning for all regions with the same data normalized. C. Hierarchical clustering of temporal-tuning profiles indicates two distinct clusters, core regions and IFG (blue) and extended regions (magenta). Asterisks indicate significant differences between ISIs at 200 msec intervals (see text). Orange asterisks indicate significant increase as presentation rates slow, blue asterisks indicate a decrease (uncorrected for multiple comparisons).

For each pair of bilateral ROIs, hemispheric asymmetries were tested via 3-way ANOVA using temporally unsmoothed data (Hemisphere [2 levels], Category [2 levels] and ISI [12 levels] as factors). As there were no significant interactions between hemisphere, ISI and category (*p*>.05), we collapsed across bilateral ROIs in subsequent analyses. The simple effect of ISI for faces allows us to identify which face-selective regions show a change in response to faces as a function of ISI. This analysis revealed modulation with ISI in all face-selective areas: core system regions, OFA, FFA and pSTS (F_(4141)_=17.6, *p*<.0001; F_(7239)_=7.0, *p*<.0001; F_(11,374)_=3.7, *p*=.0001, respectively); as well as in the extended-system areas: IFG (F_(7240)_=3.4, *p*=.002), ventro-medial PFC and precuneus (F_(7249)_=5.7, *p*<.0001; F_(11,374)_=5.2, *p*<.0001, respectively), ATL (F_(7231)_=4.3, *p*<.0001), anterior temporal face patch (ATFP; F_(11,374)_=3.3, *p*=.0002) and amygdala (F_(11,374)_=4.1, *p*<.0001). All regions survive Bonferroni correction for multiple comparisons across regions (critical alpha = 0.0055). As a secondary analysis, we considered the interaction between category and ISI, which reveals whether temporal tuning varies as a function of object category or whether temporal tuning is common for both preferred and non-preferred stimulus classes. As the ROI selection contrast (faces > places) was orthogonal to ISI, resulting estimates of category-by-ISI interactions within this ROI are unbiased (Friston et al., 2006). Normalised (Z-transformed) data were used to determine ISI-by-region interactions to ensure that significant effects were driven by differences in the true shape of the temporal tuning profile, and not potentially by temporal-tuning profiles of equivalent shape but different magnitudes in the regional response. In the core system, although the response was smaller, regional temporal tuning for the non-preferred stimulus class (places) did not strongly differentiate from that of faces. A category-by-ISI interaction showed weak evidence for differential tuning only in FFA (F_(11,374)_=1.8, *p*=.045). Temporaltuning profiles were more specific to person stimuli in extended regions, where significant category-by-ISI interactions were present in the vmPFC, ATFP and the amygdala (F_(11,374)_=1.9, *p*=.034; F_(11,374)_=1.9, *p*=.040; F_(11,374)_=2.0, *p*=.026) but did not reach significance in the precuneus, ATL or IFG (see Fig. 5). Thus, while evidence for temporal tuning within face-selective regions for face stimuli is strong (simple effect ISI for faces), temporal-tuning effects may also generalise to stimulus categories that drive regions weakly.

To formally address the patterns of interregional differences in temporal tuning apparent in Fig. 5, we performed separate pairwise 2-way ANOVAs for each combination of ROI for face stimuli (ROI [2 levels], ISI [12 levels]) using normalised beta values. With the exception of IFG, this analysis revealed strong ROI-by-ISI interactions between all core and extended regions (p-values for all pairwise regional comparisons <0.0001). Regional comparisons within the core system revealed strong dissociations in tuning between the OFA and both FFA and pSTS (p-vales <0.0001), which did not significantly differ from one another. Temporal tuning in the IFG differed from that of the OFA (*p*<0.0001), FFA (*p*=.003) and pSTS (*p*=.008), and strongly from tuning profiles in all regions of the extended system (p-values <0.0001). Reflecting the homogeneity of temporal tuning within the extended system, only a moderate difference in temporal tuning between the precuneus and amygdala was evident (*p* = 0.014), with no other significant differences in tuning being present between any other pair of extended regions.

The pairwise comparisons between ISIs (at 200 msec interval, see methods) showed the most rapid tuning in OFA, with an apparent inflection point at 400 msec, consistent with an operational time-constant of this duration (see Fig. 5). Planned 200 msec step paired t-tests showed a significant reduction in the fMRI response between 300 and 500 msec ISIs (*p*=.007) and between the following 3 couplets (400–600, 500–700, 600–800 ISIs, *p* < .0005; see Fig. 5). FFA displayed a significant increase of activity between 200 and 400 msec ISIs (*p* = .003), while the downward inflection began after 500 msec with significant reductions in activity apparent between 400 and 600 (*p* = .02), 500–700 msec (*p* = .0005), 800–1000 msec (*p* = .003) and 900–1100 (*p* < .0001) ISIs. In pSTS, activity increased from 200 to 400 msec (*p* = .03) and started its downward sweep at 500–700 msec ISIs (*p* = .002), continuing at 600–800 (*p* = .008) and 900–1100 msec ISIs (*p* = .002). Interestingly, tuning in the IFG is consistent with that of classic core regions, including the upsweep with increasing ISI (100–300, *p* = .02; 200–400, *p* = .04) and downsweep with longer ISIs initiating between 500 and 700 msec ISIs (see Fig. 5, 500–700, *p* = .005; 600–800, *p* = .0006). Taken together, this data highlights the faster tuning function in the OFA compared to other regions that showed a reduction in response at ISIs longer than 500 msec.

Regions of the extended system, except IFG, showed a slower tuning pattern compared to the core system. The ventro-medial PFC exhibited increasing responses with ISIs from 300 to 800 msec (pairwise t-tests: 300–500, 400–600, 500–700 and 600–800 msec ISIs, *p <* .001). The inflection point was reached at 800 msec ISI and decrease with longer ISIs (800–1000, *p* = .004; 900–1100, *p* = .013). The precuneus showed a similar pattern of activity, increasing its response with ISIs longer than 300 (300–500 msec, *p* = .001) and displaying a—relatively weaker—downsweep starting at 800 msec (800–1000, *p* = .01; 900–1100, *p* = .04). The ATL had a similar tuning pattern, except for the first ISI, where we observed an anomalous drop in activity (*p* = .02) before a significant enhancement between 300 and 500 msec (*p* = .001), which continued at 400–600 msec (*p* < = 0.002), 500–700 (*p* = .03) and 600–800 msec ISIs (*p* = .005), before the activity started to descend after 800 msec ISIs (800–1000, *p* = .001; 900–1100 msec, *p* = .006). The ATFP also increased its activity starting from 300 msec ISI (300–500, *p* = .0002). As with the vmPFC, precuneus and ATL, the downsweep started at 800 msec ISI (800–1000, *p* = .02). Finally, the amygdala displayed a similar increase of activity between 300 and 500 msec ISI (*p* = .002), continuing the upsweep at 600–800 msec (*p* = .02), although there was no detectable inflection point with longer ISIs in this region.

Overall, the core system exhibited an earlier and less-pronounced initial increase in responsivity with ISI than the extended system, while the subsequent decrease in responsivity with increasing ISIs occurred at longer ISIs in the extended system. The later decrease in the extended system was less pronounced than in the core and may have continued at ISIs longer than those sampled in the present experiment. Interestingly, the IFG, which is classically considered part of the extended system, has a profile similar to that of core regions. To quantify clusterings in temporal-tuning patterns across regions, hierarchical clustering analysis was performed (Fig. 5c). This analysis indicates a clear dissociation between core and extended systems and that the IFG clusters with classical core regions rather than with the extended system. In general, signal in core and extended regions increased from shorter ISIs before showing a more- or less-pronounced decrease with longer ISIs, consistent with a classic, rather than saturated, tuning model. The sole exception is the OFA. However, a reduction in response may occur at ISIs shorter than those sampled in this study, as is suggested by earlier work (McKeeff et al., 2007; Gauthier et al., 2012; Stigliani et al., 2017).

### 3.5 Place-selective regions

**Fig. 6:**
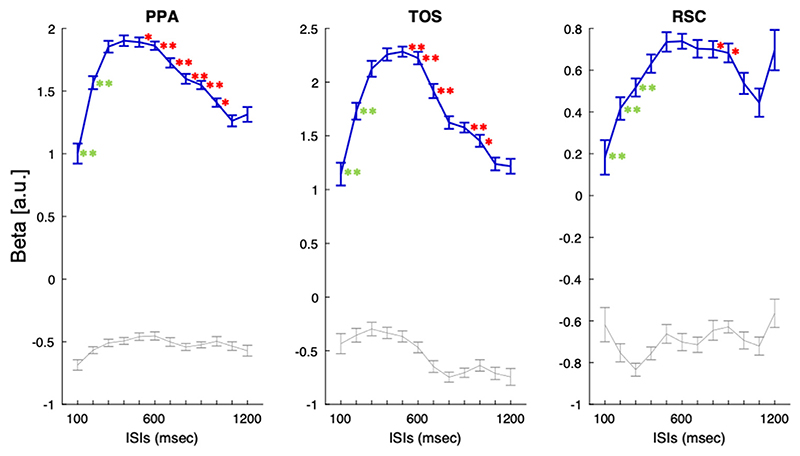
Temporal tuning in place-selective regions for place and face stimuli. Plots depict mean beta values (± 1 SEM within-subject; Cousineau, 2005) as a function of ISIs. PPA and TOS showed temporal tuning to medium presentation rates, peaking between 500 and 600 msec. RSC was suppressed at faster presentation rates and showed a maximal response at ISIs of 800 msec. The Simple Effect (SE) of ISI for each ROI are reported within each plot. Asterisks indicate significant differences between ISIs at 200 msec intervals (see text). Orange asterisks indicate significant increase as presentation rates slow, blue asterisks indicate a decrease (uncorrected for multiple comparisons).

As there was no significant interaction between hemisphere, ISI and category in place-selective regions, we collapsed ROIs bilaterally. The simple effect of ISI for place stimuli was evident in all three place-selective areas (PPA: F _(7, 229)_ = 15.1, *p* < .0001); TOS (F _(7225)_ = 12.8, *p* < .0001) and RSC (F _(11,374)_ = 3.12, *p* = .0001) as was the interaction between Category and ISI using normalised beta values: PPA (F _(11,374)_ = 5.2, *p* < .0001); TOS (F _(11,374)_ = 4.9, *p* < .0001) and RSC (F _(11,374)_ = 2.1, *p* = .019). Taken together, these results demonstrate the modulation of the fMRI response with ISI in these place-selective ROIs and the specificity of this effect to place stimuli (see Fig. 6).

To highlight the differences amongst the three areas in their responses to places stimuli, we performed separate pairwise 2-way ANOVAs for each combination of ROI (ROI [2 levels], ISI [12 levels]) using normalised beta values. Results showed that temporal tuning for place stimuli differed between RSC and both PPA (F _(7245)_ = 8.7, *p* < .0001) and TOS (F _(8261)_ = 7.4, *p* < .0001), which did not differ from one another.

Pairwise 200-msec-step t-tests showed a similar medium-tuning pattern between PPA and TOS, while RSC showed a slower tuning pattern (see Fig. 6). Both PPA and TOS displayed a similar upsweep from 100 to 300 msec (100–300, 200–400 msec, *p* < .0001) and a decrease in neural activity at ISIs longer than 600 msec (600–800, *p* = .0002 and *p*<.0001, respectively; 700–900, *p* = .0007). These two regions seemed to have a tuning pattern more similar to that of core regions. In contrast, the RSC had a tuning pattern more similar to that of the extended system. Activity increased with ISIs from 100 to 500 msec (100–300, *p* = .005; 200–400, *p* = .003; 300–500, *p* = .005) with a subtle downsweeping curve starting at ISI of 900 msec (900–1100, *p* = .02).

### 3.6 Cognitive access

**Fig. 7:**
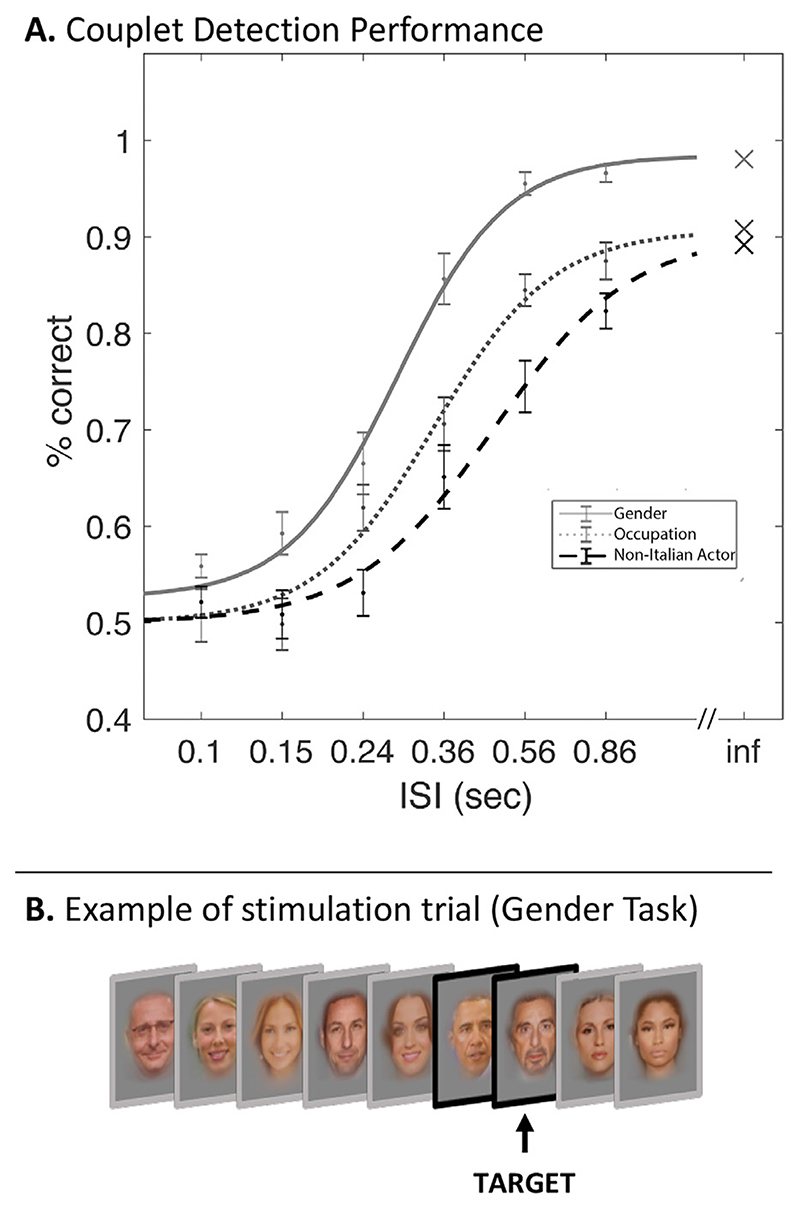
Behavioural time-constants for information access. As in the scanner experiment, participants were presented with a series of famous faces at varying ISIs. Participants had to consecutively access three levels of perceptual/semantic knowledge: Physical (gender), Binary Semantic (occupation), Combinatorial Semantic (foreign and actor). Panel A: Tuning functions for access to these three forms of perceptual/semantic knowledge. The crosses represent the ceiling performance in the unspeeded version of the experiment for each task respectively. Panel B: Example of Gender task trial. Participants had to indicate, at the end of each trial, whether a target (two consecutive males) was presented or not. This design was chosen to mirror our fMRI paradigm and circumvent reaction time delays associated with overt responses.

Activation of the extended system during one back tasks may arise due to the spontaneous retrieval of person related information (Gobbini and Haxby, 2007). To gain insight into the relationship between regional temporal-tuning properties and cognition, we compared the ISIs necessary for different levels of semantic access for each successive face presented within a sequence. The goal is to identify the minimal time required to access possible forms of information from the face stimuli and to provide some insight into the cognitive operations that may occur within the time constant of different regions delineated in the fMRI study. Fig. 7 displays behavioural estimates of the capacity to consecutively extract information of a physical (gender), binary semantic (occupation) and combinatorial semantic (non-Italian *and* actor) nature. For visual clarity, the group data has been fitted with a log-scaled sigmoid function. One-sample t-tests revealed that the capacity to extract these features (above chance performance) is already present for gender at 100 msec ISIs (t _(26)_ = 4.87, *p* < .0001) and emerges at 240 msec for occupation (t _(26)_ = 4.98, *p* < .0001), and at 360 msec for the combinatorial judgement (t _(26)_ = 4.56, *p* < .0001). These semantic features were reliably processed (passing an arbitrarily defined 80% level of performance) at 360 msec for gender, 560 msec for occupation and 860 msec for combined nationality and occupation task. To assess whether ceiling performance differed between tasks, we performed an unspeeded version of the experiment. Ceiling performance was higher for the gender task compared to both semantic tasks (paired sample *t*-test: t _(14)_ = 3.59, *p* = .003), which did not differ from one another (*t* < 1). To investigate whether lower ceiling performance was attributable to unfamiliarity with the stimuli, we reanalysed the data after removing trials where unknown personalities were targets (see methods). This did not significantly affect performance, indicating that ceiling difference reflected omissions inherent to the task rather than unfamiliarity with the stimuli.

## 4 Discussion

Timescales in the brain provide a window into hierarchical organisation and have been investigated in a variety of ways: the intrinsic autocorrelation of single units (Murray et al., 2014), integration periods of scrambled videos and narratives (Hasson et al., 2008; Lerner et al., 2011), as well as the response to visual overstimulation (Gauthier et al., 2012; McKeeff et al., 2007; Stigliani et al., 2015). Here, to understand the hierarchical nature of the distributed cortical network for perceiving and knowing about others, we probed the temporal-tuning properties across this network. Through the combination of fMRI with the presentation of images of famous people and places at varying rates (100–1200 msec), we were able to identify region-specific temporal tuning across core and extended systems. We observed different temporal tuning across regions of the cortical network for perceiving and knowing about others and a clear separation in temporal tuning between core and extended systems. A similar distinction was seen in the place-selective network; these regions showed temporal-tuning profiles similar to core (for PPA and TOS) and extended (for RSC) systems.

### 4.1 Temporal tuning in the core system

The core system (OFA, FFA, pSTS) and the IFG were tuned to more rapid face presentation than the extended system. amongst core regions, temporal tuning in OFA was distinct, as captured by the robust region by ISI interactions between OFA and other core regions. OFA exhibited comparatively stronger responses at faster ISIs and a shorter time-constant, with diminishing responses at ISIs longer than 400 msec. While the present results do not provide direct insight into the functional role of regions like OFA, and it would be wrong to draw direct conclusions about regional function based on the tuning properties of any region considered in this study, the temporal profile of OFA is nevertheless consistent with what is known from prior research about the function of this region. The short time-constant of OFA is consistent with the primacy of this region in the person-perception hierarchy (Fairhall and Ishai, 2007; Pitcher et al., 2007) and with its putative role in processing individual facial features (face-parts) as well as its relative insensitivity to higher-level facial configuration (Liu et al., 2010). Unlike OFA, in FFA, pSTS and IFG, responsiveness increases as presentation rates slow from 100 to 500 msec ISIs. The initial reduction in response may reflect partial dependence on completion of computations in OFA or, alternatively, temporal-processing capacity limits intrinsic to the dynamics of the brain regions themselves (Gauthier et al., 2012; McKeeff et al., 2007). Estimates of temporal capacity limits in FFA vary across studies (~100–400 msec), and previous studies did not observe differential tuning between OFA and FFA. Variations in temporal capacity estimates and relative insensitivity to differential tuning profiles may relate to the differing tasks and methodologies used: interspersing faces and places within blocks (Gauthier et al., 2012), alternating different or the same facial identities within blocks (Gentile and Rossion, 2014), or shorter and longer block lengths for different ISIs (Stigliani et al., 2015).

The reduced response in FFA and pSTS at ISIs longer than 500 msec indicates sufficient time for the processing of incoming stimuli within these regions (i.e. intervals longer than this are associated with a period of relative neural idleness between successive stimuli). The time-constant is notably longer than face-selective evoked potentials, such as the N170, that are believed to originate in these regions (Deffke et al., 2007; Itier and Taylor, 2004). This reflects an important difference between electrophysiological measures of the time course of the neural response and the indirect fMRI-indexed measure of neural temporal tuning used here. While the N170 captures the maximal neural response to faces, the present method measures the *duration* of the neural response. This index relies on periods of neural inactivity following presentation to produce the relative reduction in summed BOLD activity seen at the inflection point. In this way, while the N170 predicts maximal face processing in regions like the FFA at 170 msec, the current results estimate that the neural response persists until 400–500 msec. Thus, the longer time-constant observed in FFA and pSTS compared to OFA is consistent with a hierarchical increase in complexity in these regions in terms of the duration of neural processing.

Interestingly, the temporal-tuning profile of IFG closely matches core regions and was grouped with the core system, rather than the extended system, in the cluster analysis. While the IFG is traditionally grouped with the extended system (Haxby et al., 2000; Gobbini and Haxby 2007 but see also Duchaine and Yovel 2015), a link to the core system is supported by its causal role in configural face processing (Renzi et al., 2013), as well as the common pattern of recruitment of IFG and core regions when accessing different forms of person-related knowledge (Aglinskas and Fairhall, 2020), which may reflect a possible role of the IFG in the coordination of information within core regions (see also Visconti Di Oleggio Castello et al. 2017).

While the response to the non-preferred place category in core regions was notably diminished compared to the response to faces, there was not clear evidence for a dissociation in temporal-tuning profile between these stimulus categories. The lack of pronounced category-selectivity in the temporal-tuning profile may seem unintuitive, however, regions of the core system reliably exhibit a response to non-preferred categories (Downing et al., 2006), and it is possible that the weaker response to non-preferred stimulus may also be governed by the same regional temporal processing constraints evident during face processing.

### 4.2 Temporal tuning in the extended system

Regions of the extended system were tuned to slower presentation rates than core regions, with cluster analysis revealing strong partitions in the temporal tuning profiles between these systems. At faster rates, the response in extended regions was markedly supressed. As with core regions, this pattern may either reflect intrinsic properties related to the increasing complexity of the computation of these brain regions (Chen et al., 2015) or a contingency on the completion of upstream processes in core regions such as the FFA or pSTS, consistent with hierarchical organisation. The latter possibility is compatible with the traditional perceptual/non-perceptual distinction between core/extended systems and is intuitive, in that the process of perception and recognition would need to be completed prior to the automatic retrieval of person-related knowledge and the full recruitment of the extended system.

Unlike core regions, the subsequent drop in fMRI activity at longer ISIs occurred at 800 msec in the extended system. The reduction was also weaker than in the core system, and a stronger negative deflection may be apparent at ISIs longer than those we sampled. Notwithstanding the conservative nature of this 800 msec estimate, this longer time-constant is consistent with a more elaborate and complex set of cognitive computations in the extended system.

While pronounced differences were seen in tuning profiles between each extended and core system, within the extended system, tuning curves were closely aligned—both in terms of their initial upsweep and in their apparent duration. Statistically, temporal-tuning differences between regions were limited to a singular modest difference between precuneus and ATL, with no differences apparent between any of the other pairs of extended ROIs. This commonality of tuning is compatible with the sharing and coordination of information across regions of the extended system, which is in turn consistent with their documented functional profiles. The activation of the extended system by familiar faces has been attributed to the automatic retrieval of person-knowledge (Gobbini and Haxby, 2007), and these regions have been implicated in a range of diverse, but at times overlapping, cognitive processes. The ventral portion of mPFC and the amygdala are involved in facial emotion recognition (Heberlein et al., 2008; Wolf et al., 2014). The ATL, together with the ATFP, is implicated in the representation of identity information about faces (Anzellotti et al., 2014; Guntupalli et al., 2017; Wang et al., 2017). The precuneus, vmPFC and ATL are implicated in the retrieval of episodic, biographical and autobiographical memories, as well as social cognition (Burgess et al., 2001; Fairhall et al., 2014; Fairhall and Caramazza, 2013; Frith and Frith, 2007; Gesierich et al., 2012; Simmons and Martin, 2009). Indeed, accessing knowledge about other people across a variety of domains (Social, Semantic, Episodic, Physical and Nominal) commonly recruits these regions with task-related variations manifesting as subtle differences in the pattern of activation across regions (Aglinskas and Fairhall, 2020). Collectively, this previous research and the similarity of temporal-tuning profiles suggest a high degree of parallel coordination across the extended system.

### 4.3 Temporal-Tuning patterns in place-selective regions

As with person-selective brain regions, place-selective PPA, TOS and RSC also demonstrated distinct temporal-tuning properties. Although less pronounced than effects seen in the face-selective system, a more subtle dissociation was evident in the tuning to faster presentation rates of place-selective regions PPA and TOS as compared to RSC. The similarity in profile of PPA and TOS may reflect their common role in perception, as is suggested by their joint activation when viewing scenes irrespective of task (Epstein and Kanwisher, 1998) and their involvement in general scene categorisation (Dilks et al., 2011). Slower temporal tuning in RSC is consistent with a successive stage of processing. As with person-selective regions of the extended system, RSC shows a preference for familiar location as well as higher-level non-perceptual properties, such as a place’s location within its broader spatial context (Epstein et al., 2007a, Epstein et al., 2007b), autobiographical memory retrieval (Summerfield et al., 2009) or access to geographically related knowledge about people or kinds of food (Fairhall, 2020). While less clear than in face-selective regions, these results suggest the possibility that the hierarchical division into core ‘perceptual’ systems (here PPA and TOS) and extended ‘cognitive’ systems (here RSC) may be a mechanism general to object processing.

### 4.4 The relationship between time-constants and cognitive processes

While this study focussed on commonalities and differences in temporal tuning between brain regions rather than on their underlying aetiology, in this section we give some preliminary consideration to the functional implications of regional time-constants. In a behavioural study designed to mirror our fMRI experiment, we examined the time necessary to consecutively access knowledge of perceptual (gender), binary semantic (occupation) and combinatorial semantic category (actors who are not Italian). We chose the detection of couplets of stimuli rather than singletons, as this requires the full processing of the targeted feature to be completed, allowing for the subsequent correct identification of the second member of the target couplet. Perceptual categorisation reached reliable accuracy (passing the arbitrary 80% threshold) by ISIs of 360 msec, binary semantic categorisation at 560 msec ISIs and combinatorial semantic categorisation by 860 msec. For example, an ISI of 400 msec provides sufficient time for gender identification to complete but insufficient time to reliably extract semantic information. Therefore, a brain region processing the property of gender should have a time-constant less than 400 msec, while a brain region processing semantic information should have a time-constant longer than this. Relating this back to regional temporal tuning, perceptual gender categorisation could be effectively performed prior to the time-constant of the OFA, suggesting this region has sufficient computational resources to perform this operation. In contrast, basic semantic categorisation (occupation) reached reliable performance at 560 msec, well before the time-constant of extended system regions, at ISIs compatible with the time-constant of the of FFA, pSTS and IFG. While this opens the possibility that semantic information is encoded within these core regions, it is also possible that identity extraction in core regions is necessary to access semantic representations of occupation in the extended system. As discussed earlier, this possibility is supported by the observation that reliable activation in the extended system only arises as the time-constant of the core system is reached (c.f. Fig. 5). At ISIs shorter than the core systems time-constant (100-400 msec), activation is supressed in the extended system, and the categorisation of binary or combinatorial semantic couplets is either not possible or strongly impaired.

Access to combinatorial semantic knowledge (foreign actors) by 860 msec coincides with the apparent plateau of extended-system activation. In this way, the increasing response in extended regions like the precuneus or mPFC may reflect spontaneous access to fuller, combinatorial semantic knowledge, as is seen in the non-Italian actor condition. Future work will be needed to strengthen the link between behavioural and neural time-constants.

### 4.5 Load-Based temporal tuning

Assessing temporal tuning through manipulation of the interval between events sidesteps the sluggish nature of the BOLD response by capturing temporal information in the amplitude of the summed haemodynamic response to multiple events, rather than the timecourse of responses. Load-based temporal tuning is in some ways comparable to the traditional notion of time-on-task (Maus et al., 2010). By analogy, if an fMRI block were to contain many periods in which the participant is idle, this would underestimate the cortical response to a given task in comparison to a paradigm in which the participant was fully engaged throughout the block. By parametrically modulating the intertrial interval and determining the influence of this on the amplitude of the block-level BOLD response, one could discern the cognitive time required for the participant to perform the task. The present technique exploits a similar mechanism to track individual brain regions’ time-on-task and to make inferences about the duration of the neural response within a given region.

While the results of this approach are promising: delineation of temporal processing gradients within perceptual regions, clear distinctions between perceptual and non-perceptual elements of the face-processing network, and novel insight into the characteristics of the IFG, this is a relatively new and untested technique, motivating further consideration of potential concerns.

Using classic deconvolution techniques, it is not possible to accurately separate the contribution of events occurring less than two seconds apart, as the summed haemodynamic response no longer reflects the linear combination of its addends (Buckner, 1998). While block-design fMRI is normally considered robust to this limitation, it is possible that neuro-haemodynamic coupling could vary across these rapid presentation rates, with relatively attenuated BOLD responses at faster ISIs. However, work comparing the EEG and fMRI response to reversing checkerboard stimuli shows an approximately linear relationship between fMRI and EEG response at ISIs between 83 and 500 msec (Singh et al., 2003), suggesting that for block designs, the haemodynamic measures represent an unbiased estimate of neural responses at these faster ISIs. Moreover, the differential pattern of temporal tuning functions across regions suggests that variations in neuro-haemodynamic coupling are not driving the observed tuning effects.

The repetition of a stimulus is known to attenuate the fMRI response to the repeated stimulus, a phenomenon known as ‘repetition suppression’ or ‘fMRI adaptation’ (Grill-Spector et al., 2006). Adaptation effects might be expected to be minimal in the present paradigm, as stimuli changed on each presentation. However, while adaptation effects are most pronounced in the case of direct-stimulus repetition, they also occur as a result of repetition at the object or category level used in the current paradigm (Fairhall et al., 2011). Thus, it is plausible that adaptation could lead to relatively attenuated responses at faster ISIs due to the increased repetition. In contrast, within regions of the core system previously shown to produce categorical priming effects, maximal responses to stimulus presentation were observed to occur at more rapid ISIs. While this indicates that adaptation does not appear to be the main mechanism behind the observed tuning curves, it remains unknown whether adaptation effects may also be present, potentially attenuating responses at faster presentation rates.

As noted earlier, previous studies that have used rapid stimulus presentation to assess temporal processing capacity limits have produced differing estimates of processing capacity in regions like FFA (varying from ~100–400 msec). This variation underscores the importance of differing tasks and presentation sequences. For example, interspersing faces and places within blocks, which may produce underestimates of ISI effects in regions responding selectively to one or another stimulus class (Gauthier et al., 2012), and matching the total number of stimuli across ranges of ISIs (resulting in blocks lengths ranging from 2 to 8 s) does not differentiate temporal tuning across regions of the visual cortex (Stigliani et al., 2015). The use of consistent block lengths, while balancing the total number and proportion of stimulus/background across ISIs (here, 50% of the time), may contribute to the detection of the clear regional differences in temporal tuning observed in the present study.

The task used in the present study required that participants successively process each stimulus, comparing the present stimulus to the preceding stimulus. This requires full processing of each stimulus for accurate task performance. As a consequence, task difficulty increases at faster ISIs, which may influence tuning curves. However, potential task-linked effects would predict a common tuning function across cortical regions, which is inconsistent with the differentiation in tuning functions observed across regions. At the same time, using a task that does not require the full processing of each individual stimulus may obscure differential effects in the extended system, as participants may stochastically process individual stimuli while disregarding others. This would allow full processing of occasional stimuli even at rapid presentation rates, which would in turn distort the temporal-tuning function at faster ISIs.

Stimulation rates faster than a region’s computational time-constant may have produced continuous neural activity at ‘too fast’ presentation rates,’ as one stimuli are only partially processed before being interrupted by neural processing of the subsequent stimulus (saturation model), or it may produce an attenuated response (classical tuning model, c.f. Fig. 1). With the exception of OFA, all regions showed an attenuated response at more rapid ISIs, consistent with the ‘classical tuning’ model. As noted earlier, this may arise because of the temporal properties of neural response patterns inherent to the region, with reduced neural response as stimuli move away from the optimal driving stimulus in a manner analogous to that seen for orientation or motion tuning (Hubel and Wiesel, 1977; Maunsell and Van Essen, 1983). Alternatively, this may reflect contingencies in the completion of upstream processes for effective neural input into higher-order areas. As an example, higher-level extra-striate regions may not be maximally driven if the time-stimulus elaboration within V1 is insufficient. Likewise, activation of the extended system may require identity-recognition processes to conclude within core regions before identity-specific information can be accessed in the extended system. Future work will be needed to determine the relative contribution of within-region and network-level factors to attenuated regional responses at faster ISIs.

This approach is complementary to promising approaches that use multivariate representational similarity to align representation spaces across imaging modalities (Kriegeskorte et al., 2008). Similarity-based fusion of MEG and fMRI allows alignment of information spaces present in the MEG time-course with information spaces present in different brain regions as measured by fMRI, potentially providing millisecond-level resolution to regional fMRI responses (Cichy et al., 2016, Cichy et al., 2014). This is in contrast to the load-based assessment of temporal tuning employed here, which has a coarser temporal resolution. There are also differences in the type of neural activity each method may be most sensitive to. In the temporal domain, similarity-based fusion would be expected to be most sensitive to the peak of the neural response, where signal is maximal. In contrast, a load-based technique might be more sensitive to the duration of the neural response. Additionally, similarity-based approaches require reasonable heterogeneity in the stimulus classes to formulate sufficiently distinct representational spaces and could be ill-suited to stimuli that are relatively homogenous, such as famous faces.

In this study, we manipulated presentation rate to indirectly probe the temporal tuning of the network for perceiving and knowing about others: temporal properties that are not accessible with traditional non-invasive electrophysiological or fMRI approaches. We observed strong evidence for differential temporal tuning across this network, which provided insight into the organisational and hierarchical structure. Within the core system, faster temporal tuning in OFA compared to FFA and pSTS captured the hierarchical progression of these regions. The highly distinct and longer temporal-tuning pattern in extended regions is consistent with more sophisticated computations and with dependency on operations in the core, and the commonality of tuning patterns conforms to the high degree of coordination required to manifest the diverse cognitive operations accomplished by this system. Organisationally, the faster timescales in IFG indicate a functional role distinct from the rest of the extended system, which may coordinate more closely with the similarity-tuned core system. Moreover, increasing operational timescales from place-selective PPA and TOS to RSC indicates that hierarchical increases in operational timescales generalise to other forms of object processing. Collectively, these results show that regions of the person-knowledge network, as well as place-selective regions, operate over different temporal timescales consistent with hierarchical organisation.

## Figures and Tables

**Table 1:** Average peak coordinates for face- and place-selective Regions of Interest.

Region	Coordinates		
	x	y	z
Face-selective			
Right OFA	32	− 90	− 9
Left OFA	− 35	− 87	− 10
Right FFA	41	− 47	− 21
Left FFA	− 40	− 48	− 21
Right pSTS	49	− 55	13
Left pSTS	− 49	− 49	14
Right IFG	40	17	24
Left IFG	− 37	20	26
vmPFC	3	49	− 19
Precuneus	3	− 53	29
Right ATL	57	− 7	− 18
Left ATL	− 59	− 8	− 18
Right ATFP	34	− 9	− 39
Left ATFP	− 35	− 10	− 35
Right Amygdala	21	− 6	− 17
Left Amygdala	− 21	− 9	− 14
Place-selective			
Right PPA	29	− 44	− 10
Left PPA	− 27	− 47	− 9
Right TOS	35	− 81	23
Left TOS	− 31	− 82	26
Right RSC	20	− 55	16
Left RSC	− 18	− 59	17

O/FFA: Occipital/Fusiform Face Area; pSTS: posterior STS; IFG: inferior frontal gyrus; vmPFC: ventro-medial PFC; ATL: anterior temporal lobe; ATFP: anterior temporal face patch. PPA: para-hippocampal place area; TOS: transverse occipital sulcus; RSC: retrosplenial complex.

## Data Availability

Supporting data is available at: https://osf.io/rzcvb/?view_only=a82daab96fb94b92addee65a64eb309f
